# Ursolic Acid Derivative UA232 Promotes Tumor Cell Apoptosis by Inducing Endoplasmic Reticulum Stress and Lysosomal Dysfunction

**DOI:** 10.7150/ijbs.67166

**Published:** 2022-03-21

**Authors:** Wenfeng Gou, Na Luo, Bing Yu, Hongying Wu, Shaohua Wu, Chen Tian, Jianghong Guo, Hongxin Ning, Changfen Bi, Huiqiang Wei, Wenbin Hou, Yiliang Li

**Affiliations:** 1 Tianjin Key Laboratory of Radiation Medicine and Molecular Nuclear Medicine, Institute of Radiation Medicine, Peking Union Medical College & Chinese Academy of Medical Sciences, Tianjin 300192, China; 2 Center for Drug Evaluation, National Medical Products Administration, Beijing, China, 100022

**Keywords:** apoptosis, ER stress, autophagy, lysosomal membrane permeability

## Abstract

Due to increased drug and radiation tolerance, there is an urgent need to develop novel anticancer agents. In our previous study, we performed a series of structural modifications of ursolic acid (UA), a natural product of pentacyclic triterpenes, and found UA232, a derivative with stronger anti-tumor activity.* In vitro* experiments showed that UA232 inhibited proliferation, induced G_0_/G_1_ arrest, and promoted apoptosis in human breast cancer and cervical cancer cells. Mechanistic studies revealed that UA232 promoted apoptosis and induced protective autophagy via the protein kinase R-like endoplasmic reticulum kinase/activating transcription factor 4/C/EBP homologous protein-mediated endoplasmic reticulum stress. In addition, we also found that UA232 induced lysosomal biogenesis, increased lysosomal membrane permeability, promoted lysosomal protease release, and led to lysosome-dependent cell death. Furthermore, UA232 suppressed tumor growth in a mouse xenograft model. In conclusion, our study revealed that UA232 exerts multiple pharmacological effects against breast and cervical cancers by simultaneously triggering endoplasmic reticulum stress and lysosomal dysfunction. Thus, UA232 may be a promising drug candidate for cancer treatment.

## Introduction

Breast and cervical cancers are the most common malignant tumors in women, and the main cause of death in females with malignant tumors 1, 2. In recent years, the proportion of patients with these types of cancers has increased significantly, therefore it is particularly important to develop novel treatment methods to improve the quality of life of the patients and reduce the rate of tumor recurrence. The development of highly efficient, low-toxicity, and stable antineoplastic drugs has always been a topic of concern in the field of oncology.

At present, more than half of the antineoplastic drugs used in clinics come from plants and their derivatives 3. Searching for active compounds from plants, or optimizing the structure of active compounds, has become the focus of attention in the research on antineoplastic drugs worldwide. Pentacyclic triterpenes are natural compounds that are widely present in nature. Studies have shown that saponins containing pentacyclic triterpenes have extensive pharmacological effects and important biological activities, such as anti-inflammatory, liver protection, and anti-tumor, activities, which provide a reliable experimental basis for the development and utilization of pentacyclic triterpenes 4-6.

Ursolic acid (UA) is a pentacyclic triterpenoid, which is widely found in many plants 7. UA is widely used in the pharmaceutical industry because of its high medical value and low side effects. It has many biological effects, such as sedation, anti-inflammation, liver protection, and anti-tumor effects 8-10. Studies have shown that UA shows clear anti-tumor activity in a variety of malignant tumors, such as liver, colon, breast, cervical cancers 11-14. It can inhibit the growth of tumor cells and induce autophagy and apoptosis 15. Although UA has a wide range of biological activities, its poor water solubility, and low bioavailability limit its clinical application 16. To overcome these shortcomings, researchers have designed and developed a large number of UA derivatives with novel structures and strong activities via chemical modifications of the UA matrix.

Our research group carried out a series of modifications to UA and screened out the derivative, UA232, that shows strong anti-tumor activity 17. It was found that its anti-tumor effect on lung cancer was significantly stronger than that of UA. To further explore its broad-spectrum effect, we selected female malignant tumors, breast and cervical cancers for in-depth study and systematically revealed the anti-tumor mechanism of UA232.

## Materials and methods

### Reagents and antibodies

UA232 was synthesized in our laboratory with a purity of > 98%. UA, 3-methyladenine (3-MA), and CA-074 methyl ester (CA-074-me) were purchased from MedChemExpress (Monmouth Junction, NJ, USA), and 4-phenylbutyric acid (4-PBA) was purchased from Energy Chemical (Shanghai, China). Crystal violet, 4',6-diamidino-2-phenylindole dihydrochloride (DAPI), 4% paraformaldehyde fix solution, Lyso-Tracker Red dye, and Cell Counting Kit-8 (CCK-8) were purchased from Beyotime Biotechnology (Shanghai, China). The cell cycle detection kit and Annexin V-FITC/PI kit were purchased from KeyGen Biotech (Nanjing, China). Primary antibodies against cyclin D1, CDK4, Bax, Bcl-2, caspase-8, caspase-3, PARP1, CHOP, GRP78, ATF4, LC3, P62, and LAMP2 were purchased from Proteintech Group (Rosemont, IL, USA). Antibodies against PERK, p-PERK, eIF2a, and p-eIF2a were purchased from Bioss (Beijing, China). Antibodies against cathepsin B were purchased from Abcam (Cambridge, MA, USA). β-actin, horseradish peroxidase conjugated secondary antibodies (goat anti-mouse or goat anti-rabbit), and fluorescently labeled secondary antibodies (IgG Alexa Fluor® 488 or 594 goat-anti-rabbit) were obtained from ZSGB BIO (Beijing, China). Trizol reagent, cDNA synthesis kit, and UltraSYBR mixture were purchased from CoWin Biotech (Beijing, China).

### Cell lines and cell culture

MCF7, MDA-MB-231, ME-180, HeLa, and HIEC-6 cells were purchased from the American Type Culture Collection (ATCC) and cultured in DMEM medium supplemented with 10% fetal bovine serum and 1% penicillin-streptomycin in a humidified atmosphere of 5% CO_2_ at 37 ℃.

### Cell viability assay

CCK-8 assay was used to test the viability of cells treated with UA or UA232. Briefly, the cells (3000 cells/well) were cultivated in a 96-well plate and cultured for 24 h. Different concentrations (2.5-50 µM) of UA or UA232 were used to treat cells for 24, 48, or 72 h. Then CCK-8 solution was added and incubated for 2 h in a CO2 incubator. The absorbance of the final solution was measured at 450 nm using a TECAN Infinite 200 (Männedorf, Switzerland).

### Colony formation assay

Colony formation assay was used to determine the effects of UA232 and UA on the proliferation of MCF7 or HeLa cells. Cells were counted and seeded in a 6-well plate at a density of 5000 cells/well and incubated overnight. The cells were treated with UA232 or UA for 24 h. The growth medium was then replaced with fresh medium. The seeded cells were cultured for two weeks and then fixed with 4% paraformaldehyde for 30 min. After washing twice with phosphate-buffered saline (PBS), the cells were stained with crystal violet for 15 min, and the colonies were photographed.

### Flow cytometry analysis of the cell cycle

MCF7 or HeLa cells were treated with UA232 (4 or 6) μM for 12, 24, and 48 h and then fixed with ice-cold 70% ethanol overnight at -20 ℃. The fixed cells were washed twice with PBS and stained with propidium iodide (PI) working solution containing RNase in the dark at room temperature for 30 min. The stained cells in different phases of the cell cycle were determined with FACScan flow cytometry. Data were then analyzed using the ModFit LT software.

### Flow cytometry analysis of cell apoptosis

The cells were directly treated with different concentrations of UA or UA232 for 12 h or pretreated with 3-MA (5 mM), 4-PBA (2.5 mM), CA-074-me (5 µM) for 2 h before administration. Staining was performed according to the instructions provided in the Annexin V-FITC/PI staining kit. FACScan flow cytometry was used to analyze cell apoptosis. Data were analyzed using BD Accuri C6 software.

### Protein extraction and western blotting

MCF7 or HeLa cells were treated with different concentrations of UA232 for 12, 24, or 48 h and then lysed in ice-cold radioimmunoprecipitation assay (RIPA) lysis buffer supplemented with a protease inhibitor, phenylmethanesulfonyl fluoride (PMSF). Concentrations of all samples were determined and diluted to the same level according to the instructions provided in the BCA Protein Assay Kit. Equal amounts of proteins were loaded onto the sodium dodecyl sulfate-polyacrylamide (SDS-PAGE) gel and transferred onto a polyvinylidene fluoride (PVDF) membrane. The PVDF membrane was then immersed in 5% skim milk powder at room temperature for 1 h and incubated in a refrigerator at 4 ℃ with the specific primary antibody overnight. After washing thrice with PBS containing 0.1% Tween-20 for 5 min each, the PVDF membrane was incubated with the corresponding secondary antibody for 2 h at room temperature. After washing thrice with PBS, the proteins were detected using a chemiluminescence method. All bands were normalized to β-actin as an internal control. Quantification of band density was performed using the Scion Corporation software.

### Immunofluorescence staining

After being treated with different concentrations of UA232 for 12 h, the cells were fixed with pre-cooled 4% paraformaldehyde at 4℃ for 15 min and then washed twice with PBS. The fixed cells were incubated in 0.1% Triton X-100 in PBS for 30 min and washed twice with PBS. The cells were blocked in 5% BSA for 30 min, washed twice with PBS, and incubated overnight with the specific primary antibody at 4 ℃. The cells were then washed thrice with PBS and incubated with a goat anti-rabbit or anti-mouse secondary antibody for 2 h at 25 ℃. The operation of the second protein in the co-localization immunofluorescence experiment was the same as that described above. Cells were finally stained with DAPI staining solution for 5 min, washed thrice with PBS, and fluorescence images were visualized under a confocal microscope.

### Total RNA extraction and real-time polymerase chain reaction (PCR)

After treatment with different concentrations of UA232 for 12 h, total RNA was isolated using Trizol reagent, according to the manufacturer's instructions. Reverse transcription of total RNA was performed using a cDNA kit. The primer sequences were as follows: GAPDH-forward: 5′-CAATGACCCCTTCATTGACC-3′ and GAPDH-reverse: 5′-TGGAAGATGGTGATGGGATT-3′; TFEB-forward: 5′-GGTGTTGAAGTTGGATGATGTC-3′ and TFEB-reverse: 5′-GCATCTGCATTTCA GGATTGAT-3′; LAMP1-forward: 5′-CTCTGTGGACAAGTACAACGT-3′ and LAMP1-reverse: 5′-GTTGATGTTGAGAAGCCTTGTC-3′; LAMP2-forward: 5′-CAGAATGGTCCCAAAATAGCAG-3′ and LAMP2-reverse: 5′-TTCAGCATCAG GAAATTTGTG-3′; CTSB-forward: 5′-ATACTCAGAGGACAGGATCACT-3′ and CTSB-reverse: 5′-ATCTTTTCCCAGTACTGATCGG-3′; CTSD-forward: 5′-GTACATGATCCCCTGTGAGAAG-3′ and CTSD-reverse: 5′-ACGGTCAAACACAGTG TAGTAG-3′. Quantitative real-time PCR was performed to determine the mRNA levels of the specific genes. The 2^-ΔΔCT^ method was used to calculate the fold change. The results were normalized to that of the internal control (GAPDH).

### *In vivo* efficacy experiments

HeLa cells (5 × 10^6^ cells) were subcutaneously injected into the flanks of 4-week-old female BALB/c nude mice (HFK Bioscience Co, Beijing, China). Tumor volume was measured with a digital caliper every 2 days, tumor volume (mm^3^) = (length) × (width)^2^ × π/6. When the tumor volumes reached 100 mm^3^, the mice were randomized into 4 groups (n = 5) that received the following: model, UA (100 mg/ kg), and UA232 (50 or 100 mg/kg). Mice were intraperitoneal injection these drugs every 2 days for a total of 7 times. The body weight and tumor volume were recorded every 3 days. At the end of the experiment, all of the tumors and organs were collected and measured. All animal experiments were approved by the Experimental Animal Ethics Committee of the Institute of Radiation Medicine (IRM-DWLL-2020055) and compliant with the Guide for the Care and Use of Laboratory Animals.

### Statistical analysis

Data were expressed as the mean ± SD from three independent experiments and evaluated by one-way analysis of variance (ANOVA) using GraphPad Prism 8.0.1 software or IBM SPSS Statistics 22.0 software (SPSS, USA). Differences were considered significant at p < 0.05.

## Results

### UA232 inhibited the proliferation of MCF7 and HeLa cells by inducing G_0_/G_1_ phase arrest

**Figure 1:**
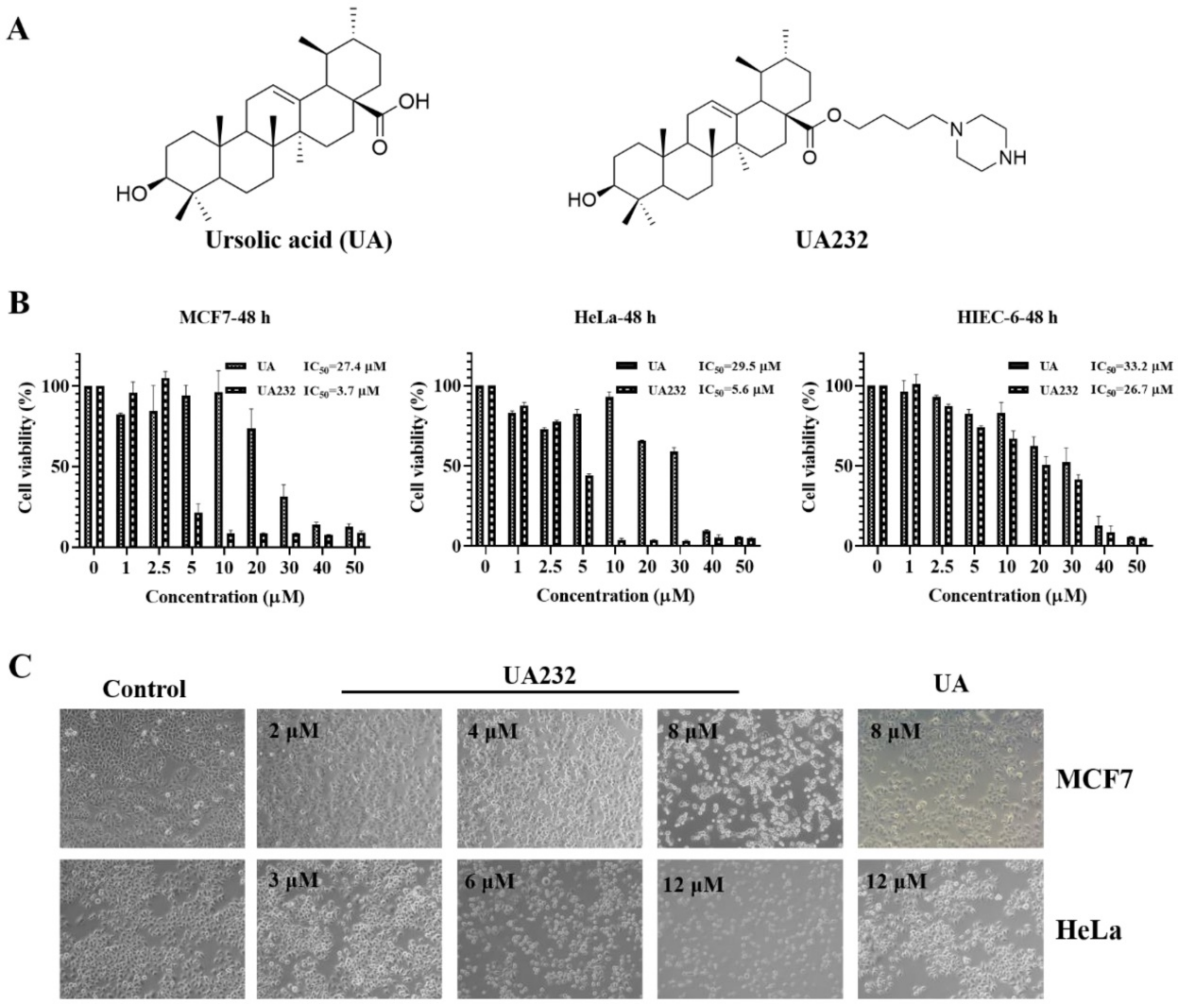
** Effects of UA232 on the viability of MCF7 and HeLa cells. (A)** Chemical structures of ursolic acid (UA) and UA232.** (B)** Cell inhibition rate was determined by CCK-8 assay in MCF7, HeLa, or HIEC-6 cells treated with UA232 or UA (0-50 μM) for 48 h. **(C)** Cellular morphologies of MCF7 or HeLa cells were detected after treatment with the indicated concentrations of UA232 or UA for 24 h.

We used the CCK-8 assay to validate the cytotoxic effects of UA232 on MCF7, MDA-MB-231, ME-180, and HeLa cells. The results showed that UA232 decreased the viability of MCF7, MDA-MB-231, ME-180, and HeLa cells in a dose-dependent manner, and its anti-tumor effect was significantly stronger than that of UA, while the cytotoxicity of UA232 to human normal cell HIEC-6 was similar to that of UA (Fig. 1B, Fig. S1A-B). Morphological observations showed that the cells in the control group of MCF7 and HeLa cells were normal and grew in clusters. With the increase in UA232 concentration, the cell density decreased gradually, and the cells lost their normal morphology. At 8 μM (MCF7) or 12 μM (HeLa), the cells completely lost their normal shape and became round or even ruptured (Fig. 1C).

**Figure 2:**
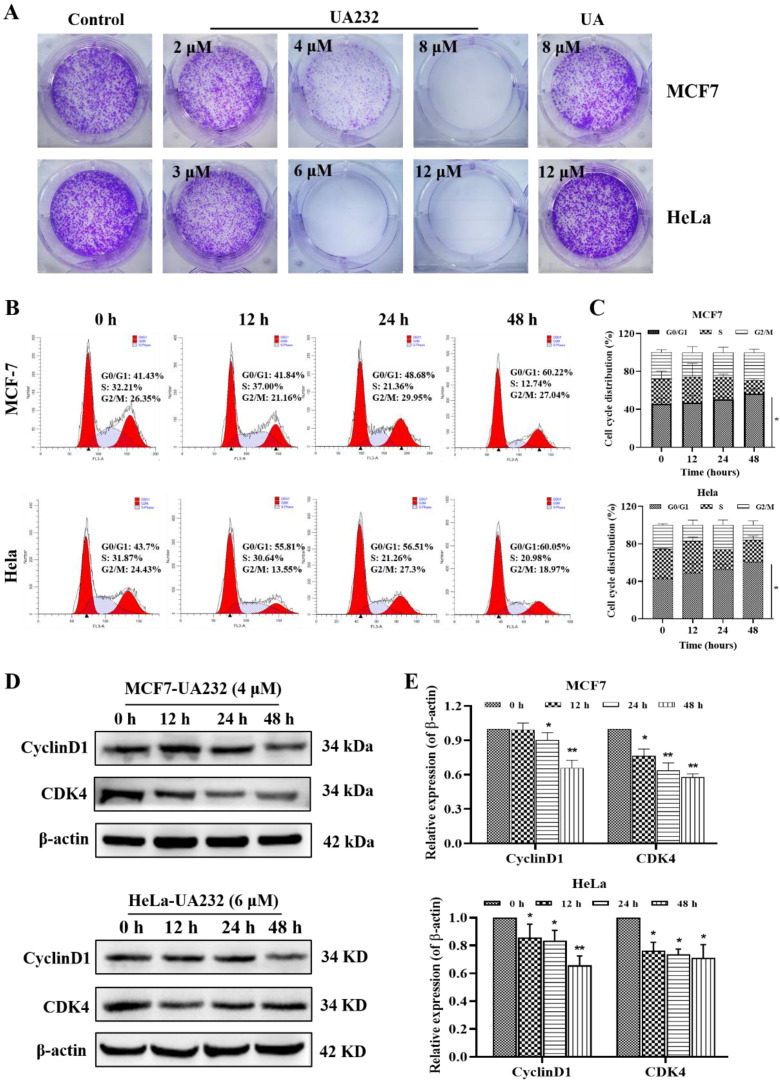
** UA232 arrests cell cycle in MCF7 and HeLa cells. (A)** Colony-forming abilities of the MCF7 or HeLa cells were measured after UA or UA232 treatment for 24 h and continued culture for two weeks. **(B)** Cell cycle distribution of MCF7 and HeLa cells treated with UA232 (4 or 6 μM) for the indicated time (0, 12, 24, and 48 h) was measured by flow cytometry with propidium iodide (PI) staining. **(C)** Percentage of cells in specific cell cycle phase was quantified in panel B. **(D)** Expression levels of G_0_/G_1_ phase-related proteins in MCF7 or HeLa cells treated with UA232 (4 or 6 μM) for 0-48 h were analyzed by western blotting. **(E)** Quantitative data of western blotting analysis in panel D. These experiments were repeated at least three times. ^*^p < 0.05 versus control; ^**^p < 0.01 versus control.

To further explore the effect of UA232 on the proliferation of MCF7 and HeLa cells, we analyzed the regulatory effect of UA232 on the HeLa cell cycle by colony formation assay and flow cytometry. These results indicated that UA232 inhibited the ability of cell colony formation (Fig. 2A). We found that UA232 arrested the cells in the G_0_/G_1_ phase in a time-dependent manner (Fig. 2B-C). Western blotting analysis indicated that after treatment with UA232 (4 or 6 μM), the abundance of cyclin D1 and CDK4, which play important roles in cell cycle regulation, were significantly reduced in a time-dependent manner (Fig. 2D-E). These results demonstrated that UA23 inhibits the proliferation of HeLa and MCF7 cells by inducing G_0_/G_1_ arrest.

### UA232 induced apoptosis in MCF7 and HeLa cells

**Figure 3:**
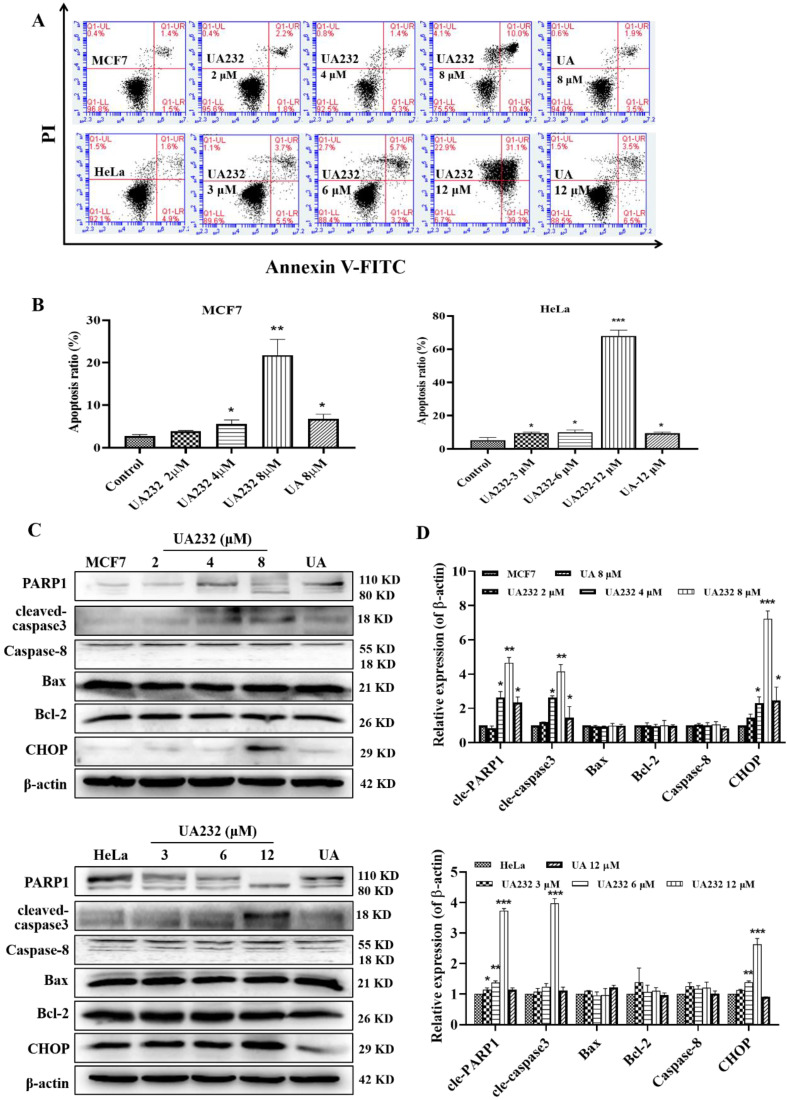
**UA232 induces apoptosis in MCF7 and HeLa cells. (A)** Apoptosis of MCF7 or HeLa cells treated with different concentrations of UA232 or UA for 12 h were measured by flow cytometry with Annexin V-FITC staining. **(B)** Percentage of apoptosis in MCF7 or HeLa cells was quantified in panel A. **(C)** Changes in the expression levels of cell apoptosis-related proteins in MCF7 or HeLa cells treated with different concentrations of UA232 or UA for 12 h. **(D)** Quantitative data of western blotting analysis in panel C. These experiments were repeated at least three times. ^*^p < 0.05, ^**^p < 0.01, ^***^p < 0.001 versus control.

Cell morphological observations showed that UA232 could induce cell death. To study the mechanism of cell death induced by UA232, we first detected the occurrence of apoptosis by Annexin V-FITC/PI staining. Since MCF7 or HeLa cells were fragmented and unable to collect cells after 12 h of UA232 (8 or 12 μM) treatment, we collected cells treated with UA232 for 12 h for protein detection (Fig. S1C). The results showed that apoptosis increased significantly after cells were treated with UA232 for 12 h in a dose-dependent manner, indicating that UA232 has an anti-tumor effect by inducing apoptosis in MCF7 and HeLa cells (Fig. 3A-B). Studies have shown that multiple pathways regulate apoptosis 18. To further study the main pathways of apoptosis induced by UA232, we detected the marker proteins of the main pathways by western blotting. As shown in Fig. 3C, consistent with the phenotype observed, the results of western blotting revealed that with the increase in UA232 concentration, cleaved-PARP and cleaved-caspase3, which are the classical characteristic of apoptosis, gradually accumulated, while the expression levels of Bcl2, Bax, and caspase-8 did not change significantly. These results suggest that UA232-induced apoptosis is not related to the endogenous pathway of mitochondrial apoptosis and the exogenous pathway of the death receptor (Fig. 3C-D). Interestingly, we found a significant increase in the expression levels of CHOP (Fig. 3C-D). CHOP is considered an important apoptosis factor in ER stress-induced apoptosis, suggesting that UA232-induced apoptosis may be related to ER stress.

### UA232 induced apoptosis via regulation of the PERK/eIF2α/ATF4-mediated ER stress

**Figure 4:**
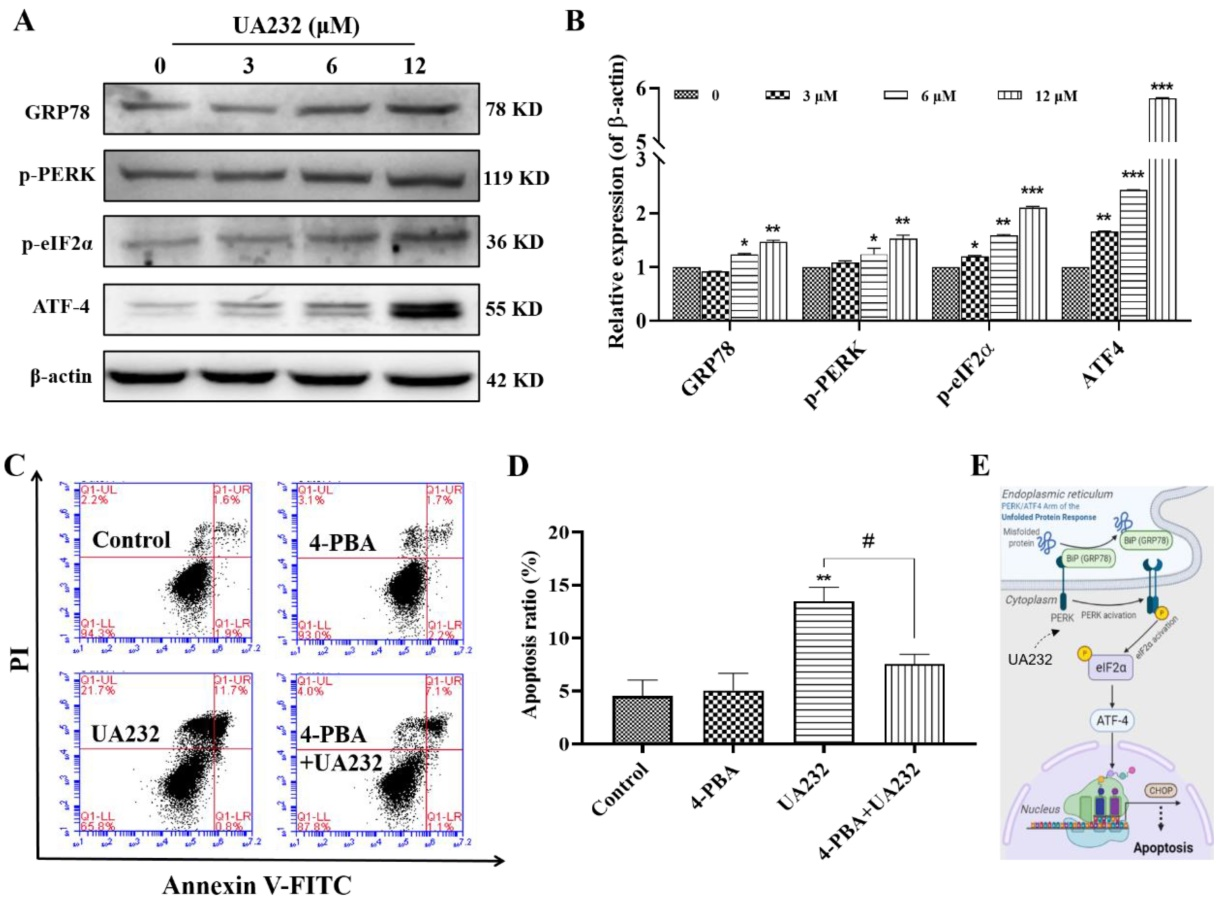
**UA232 triggers endoplasmic reticulum (ER) stress leading to apoptosis. (A)** Changes in the expression levels of ER stress-related proteins in HeLa cells treated with different concentrations of UA232 for 12 h. **(B)** Quantitative data of western blotting analysis in panel A. **(C)** Apoptosis of HeLa cells treated with UA232 (6 μM), 4-phenyl butyric acid (4-PBA, 2.5 mM), or both for 12 h was measured by flow cytometry with Annexin V-FITC staining. **(D)** Percentage of apoptosis in HeLa cells was quantified in panel C. **(E)** Schematic representation of mechanisms of UA232 regulated apoptosis *in vitro*. These experiments were repeated at least three times. ^*^p < 0.05, ^**^p < 0.01 versus control;*^ #^p* < 0.05 versus UA232.

To further confirm the ER stress induced by UA232, we detected the expression levels of ER stress marker, heat shock protein family A member 5 (HSPA5/ GRP78), and other related proteins. As shown in Fig. 4A-B, GRP78, p-PERK and p-eIF2a were significantly upregulated. The expression levels of ATF4 downstream of eIF2a were also significantly increased, indicating that UA232 induced ER stress. Although the principal aim of the unfolded protein response (UPR) is to restore ER homeostasis, if ER stress is irreversible, ATF4-CHOP activation can induce the apoptotic pathway 19. To further prove that ER stress promoted apoptosis, we selected the ER stress inhibitor 4-PBA for further analysis. The results showed that the pro-apoptotic effect of UA232 was partly reversed by inhibition of ER stress using 4-PBA (Fig. 4C-D). These results confirmed that UA232 induced ER stress-mediated apoptosis, which was regulated by the PERK/ATF4/CHOP pathway (Fig. 4E).

### UA232 activated protective autophagy in MCF7 and HeLa cells

**Figure 5:**
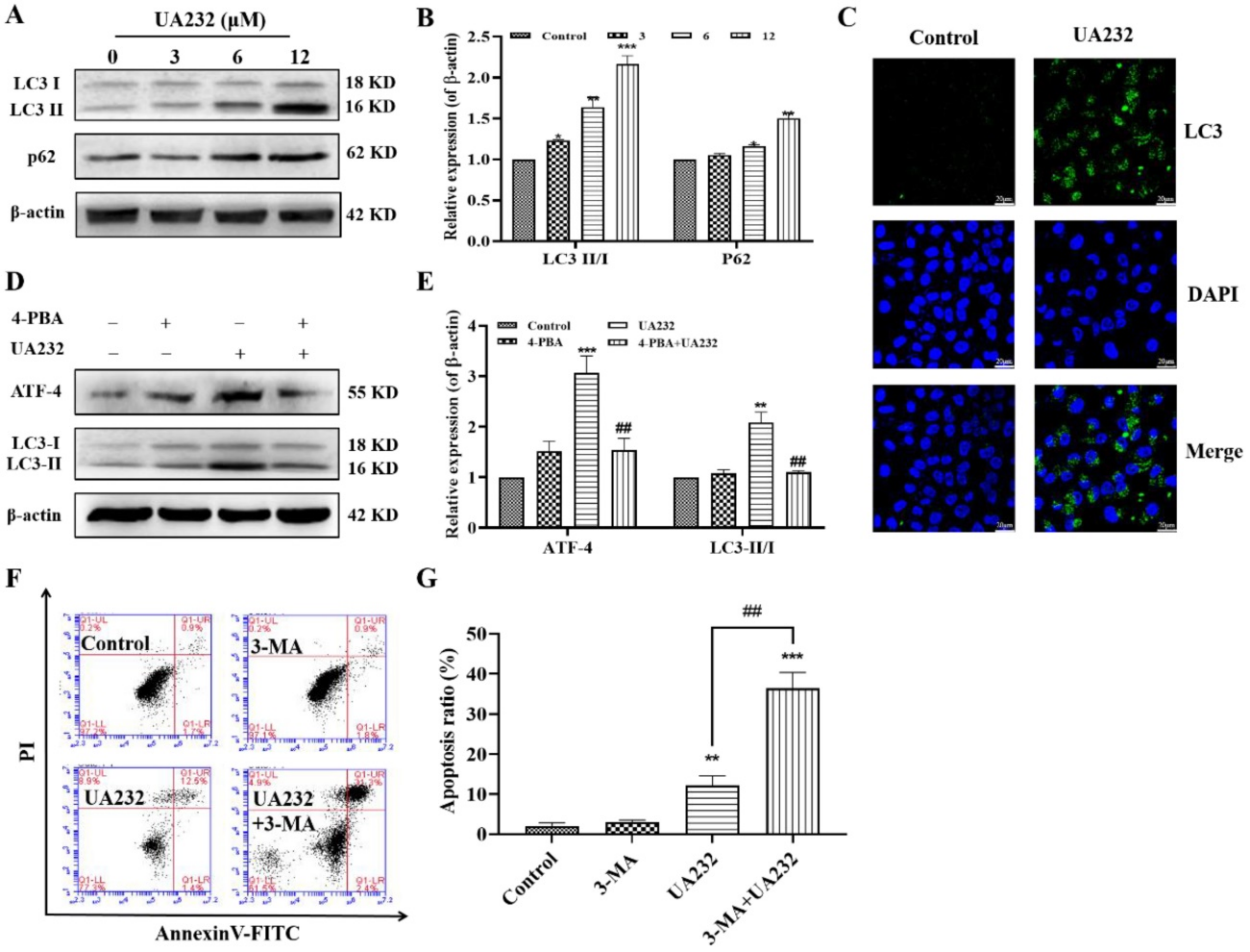
** UA232 induces protective autophagy. (A)** Changes in the expression levels of autophagy-related proteins in HeLa cells treated with different concentrations of UA232 (3, 6, or 12 μM) for 12 h. **(B)** Quantitative data of western blotting analysis in panel A.** (C)** Immunofluorescence staining of LC3 in HeLa cells treated with or without UA232 (6 μM). Nuclei are indicated in blue color (DAPI) staining. Scale bar = 20 μm.** (D)** Expression levels of ATF4 and LC3-II/I in HeLa cells treated with UA232 (6 μM), 3-MA (5 mM), or both for 48 h.** (E)** Quantitative data of western blotting analysis in panel D.** (F)** Apoptosis of HeLa cells treated with UA232, 3-methyladenine (3-MA), or both for 12 h was measured by flow cytometry with Annexin V-FITC staining.** (G)** Percentage of apoptosis in HeLa cells was quantified in panel F. ^*^p < 0.05,^ **^p < 0.01,^ ***^p < 0.001 versus control; ^##^p < 0.01 versus UA232.

The above results suggest that the inhibition of ER stress does not completely reverse UA232-induced apoptosis, indicating the involvement of other pathways. ER stress has been reported to induce autophagy 20. The conversion of LC3-I to LC3-II by the addition of phosphatidylethanolamine is essential for the formation of autophagosomes and can serve as a marker of autophagy 21. To investigate whether UA232 also triggered autophagy in MCF7 and HeLa cells, western blotting analysis was used to measure the expression levels of LC3. We found that the LC3-II/LC3-I ratio increased with an increase in UA232 concentration, indicating that UA232 triggered the accumulation of autophagosomes in MCF7 and HeLa cells in a dose-dependent manner (Fig. 5A-B, Fig. S2A-B). We further detected the expression of LC3 by immunofluorescence *in situ*, and as we expected, UA2332-treated MCF7 or HeLa cells showed higher LC3 expression levels than the control group (Fig. 5C). To explore the relationship between autophagy and the ER, we selected ER stress inhibitor 4-PBA for further study. Western blotting results showed that 4-PBA pretreatment for 2 h and then incubation with UA232 (6 μM) resulted in a recovery of LC3-II expression (Fig. 5D-E), indicating that UA232-induced autophagy was regulated by ER stress. In addition, to determine the role of UA232-induced autophagy in cell survival, we selected the autophagy inhibitor 3-MA to further analyze the changes in apoptosis. As shown in Fig. 5F-G, inhibition of autophagy increased apoptosis in HeLa cells, indicating that UA232-induced autophagy is involved in protective autophagy.

### UA232 promoted lysosomal biogenesis and induced lysosomal membrane permeabilization

**Figure 6:**
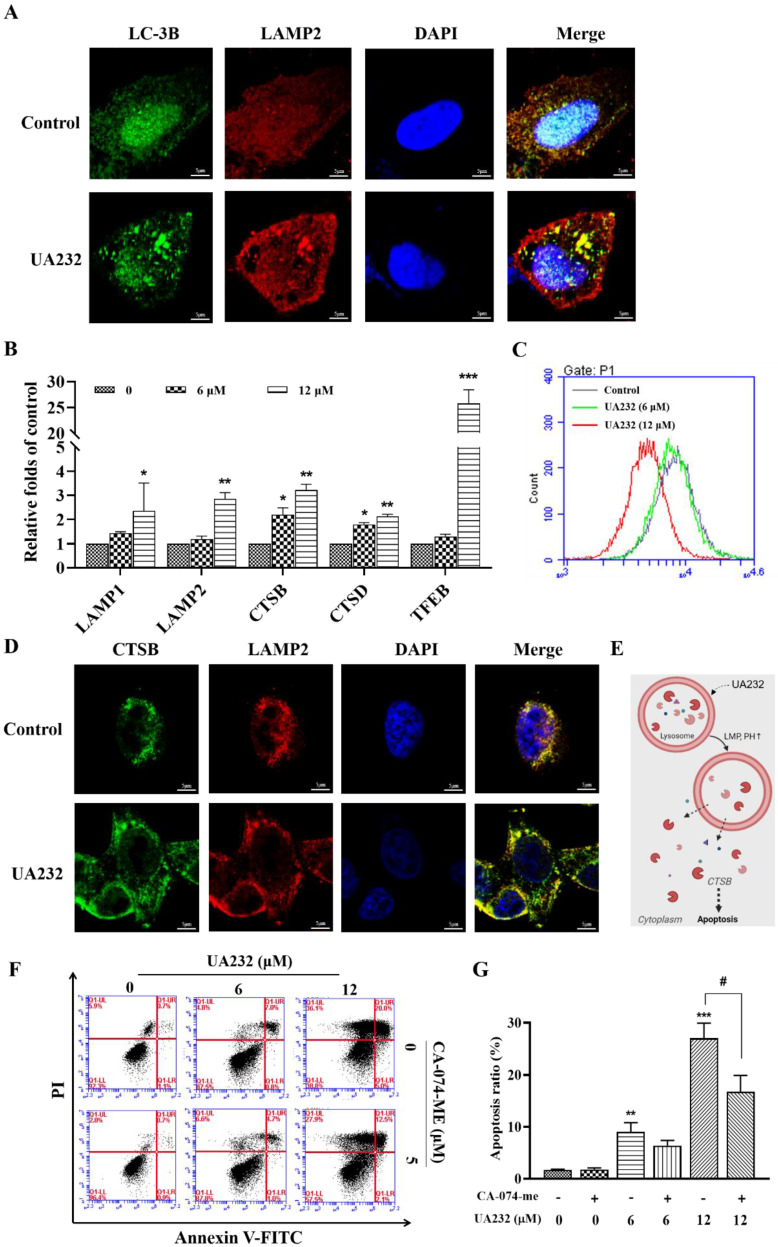
** UA232 induces lysosomal dysfunction resulting in the inhibition of autophagic flux. (A)** Immunofluorescence staining of LC3 (green) and LAMP2 (red) in HeLa cells treated with or without UA232 (6 μM). Scale bar = 5 μm. **(B)** mRNA expression levels of lysosomal biogenesis-related genes (LAMP1, LAMP2, CTSB, CTSD, TEFB) in HeLa cells treated with different concentrations of UA232 (6 or 12 μM) for 12 h. **(C)** Lyso-Tracker Red labeling lysosome and flow cytometry were used to detect the changes in lysosome acidity. **(D)** Immunofluorescence staining of CTSB (green) and LAMP2 (red) in HeLa cells treated with or without UA232 (6 μM). Scale bar = 5 μm. **(E)** Schematic representation of the mechanisms of UA232 regulated apoptosis *in vitro*. **(F)** Apoptosis of HeLa cells treated with UA232, CA-074-ME (5 μM), or both for 12 h was measured by flow cytometry with Annexin V-FITC staining. **(G)** Percentage of apoptosis in HeLa cells was quantified in panel F. ^*^p < 0.05, ^**^p < 0.01, ^***^p < 0.001 versus control; ^#^*p* < 0.05 versus UA232.

Interestingly, western blotting results showed that the expression levels of P62, a marker of autophagy degradation, also increased (Fig. 5A). Moreover, the observation of cell morphology showed that vacuole-like changes occurred in cells treated with high-dose UA232 (Fig. S3). We speculate that UA232 may block autophagic flux. To verify this conjecture, we used an immunofluorescence assay to detect the co-localization of autophagy protein LC3 and lysosomal protein LAMP2 to further observe the change in autophagy flux. As shown in Fig. 6A, a large number of yellow puncta could still be observed in cells treated with UA232 (12 µM) suggesting that UA232 did not prevent the fusion of autophagosomes and lysosomes. Interestingly, we noticed a translocation of lysosomes from the nucleus to the plasma membrane after treatment with UA232 (Fig. 6A).

It has been reported that lysosomal accumulation of hydrophobic weak base drugs can activate TFEB, resulting in the transcription of its downstream genes 22. TFEB is a master transcriptional regulator of lysosomal biogenesis and function 23. RT-PCR results showed that the mRNA levels of TFEB and its downstream genes, including LAMP1, LAMP2, CTSB, and CTSD, increased significantly in a dose-dependent manner, indicating that UA232 promotes lysosomal biogenesis (Fig. 6B). Next, we explored whether UA232 caused lysosomal dysfunction. Lysotracker Red Staining is a probe that can stain acidic organelles with red fluorescence and is used to monitor pH in lysosomes. According to the results of flow cytometry, we found that the fluorescence intensity in cells treated with UA232 was decreased, which indicated that the pH value in lysosomes was increased by UA232 (Fig. 6C).

Immunofluorescence analysis was used to further analyze whether UA232 triggers lysosomal membrane permeabilization (LMP). As shown in Fig. 6D, there was diffuse green fluorescence in the cells treated with UA232, indicating that UA232 induced LMP, resulting in the infiltration of CTSB into the cytoplasm (Fig. 6E, Fig. S4). Lysosome damage and internal protease release can lead to apoptosis. CTSB is a typical lysosomal cysteine protease that is involved in apoptosis 24. To investigate whether lysosomal protease release is involved in UA232-induced apoptosis, we used the selective CTSB inhibitor CA-074-Me for further exploration. We found that the apoptosis rate of cells treated with UA232 combined with CA-074-Me was lower than that of cells treated with UA232 alone (Fig. 6F-G). These results suggest that UA232 promotes the biogenesis of lysosomes, increases the membrane permeability of lysosomes, leads to the infiltration of CTSB into the cytoplasm and induces cell death.

### UA232 inhibited tumor growth *in vivo*

**Figure 7:**
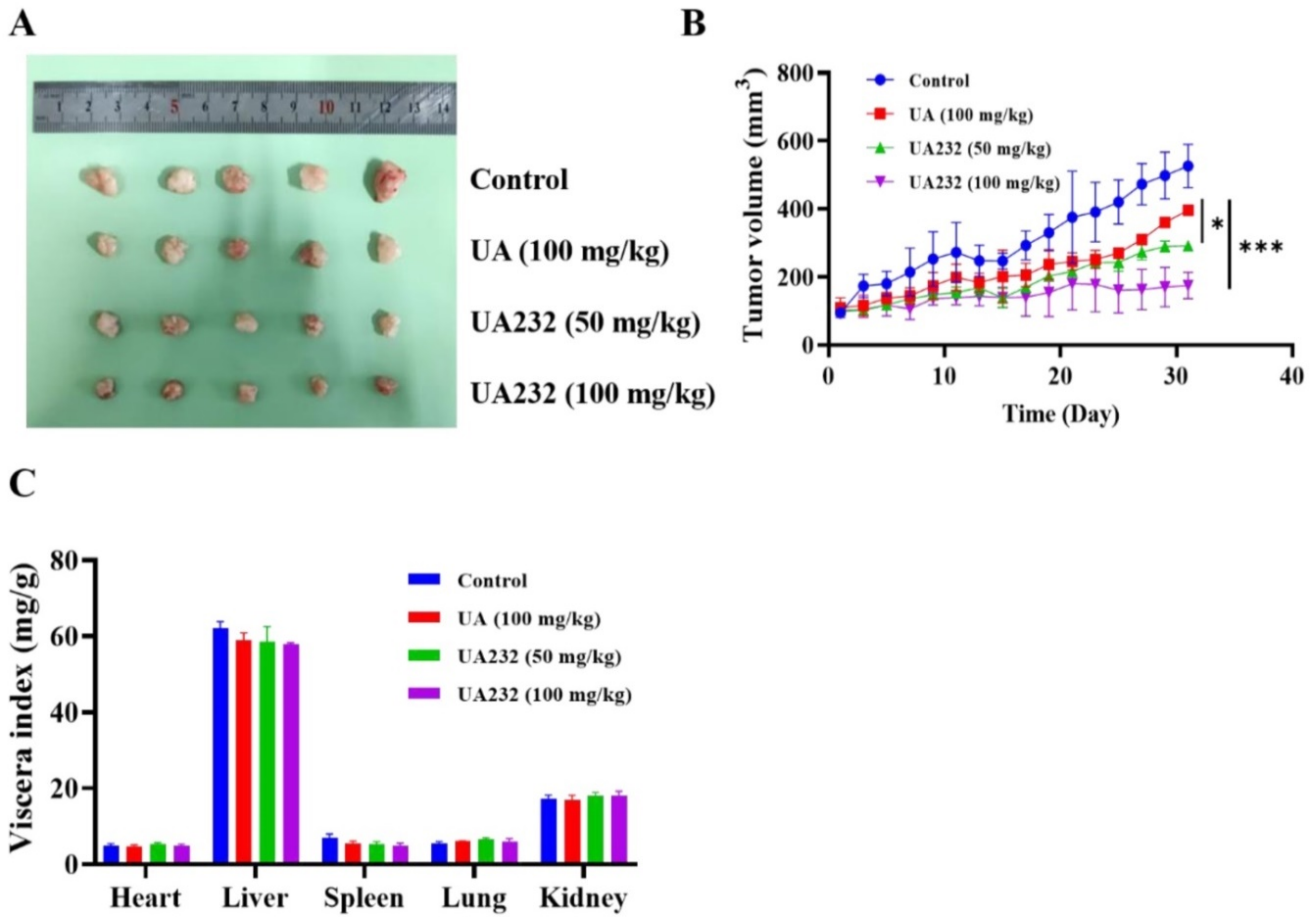
** UA232 induces a potent anti-tumor effect *in vivo*. (A)** Images of resected HeLa cell nude-mouse xenograft tumor samples. **(B)** Tumor growth curves were determined by measuring the tumor volume. The bars indicate the mean ± SD (n = 5). **(C)** The viscera index of the main organs of mice was isolated from the treated and untreated groups to evaluate the toxicity of UA232. ^*^p < 0.05, ^***^p < 0.001 versus UA.

To further investigate the anti-tumor effect of UA232, we performed xenograft experiments to study whether UA232 inhibited tumor growth *in vivo*. The results showed that both UA and UA232 could inhibit tumor growth, and the effect of UA232 at a low dose of 50 mg/kg was better than that of UA (100 mg/kg) (Fig. 7A, B). Additionally, according to the viscera index analysis, no differences were observed in the heart, liver, spleen, lung, or kidney of the UA232-treated group compared to the control group (Fig. 7C). All of these results indicate that UA232 exhibits a potential anti-tumor effect and low toxicity *in vivo*.

## Discussion

In this study, we found that UA232 has a good anti-tumor effect *in vitro* and *in vivo*, which by inhibiting cell proliferation, inducing G_0_/G_1_ phase arrest, and promoting apoptosis. Apoptosis is usually divided into intrinsic and extrinsic pathways 25. We used western blotting to detect the expression levels of Bcl-2 and Bax, two of which represent the intrinsic pathway of mitochondrial apoptosis, and the expression levels of Caspase-8 which represents the extrinsic pathway of death receptor apoptosis 26. The results showed that UA232 did not promote apoptosis through these classical pathways.

Increasing evidence suggests that ER stress may play a role in the cytotoxicity of many natural compounds 27-29. ER stress was originally a protective response to different types of cellular damage. However, under severe and sustained ER stress conditions, the UPR mediated by ER stress might fail to re-establish ER homeostasis and switch to pro-death mechanisms 30. If ER stress is irreversible, the activated ATF4-CHOP pathway can induce the apoptotic pathway 19. CHOP is a critical protein in ER stress-mediated apoptosis that possesses the ability to promote DNA damage and inhibit cell growth. In this study, the expression levels of CHOP were increased by UA232 in a dose-dependent manner. We found that the PERK-ATF4 pathway, a branch of the UPR pathway, was activated. Moreover, the pro-apoptotic effect of UA232 can be partly reversed by inhibition of ER stress using 4-PBA which reflects the fact that the apoptosis induced by UA232 is close to the ER stress and UPR.

Autophagy is a dynamic process that consists of four consecutive stages: isolation membrane, vesicle elongation, autophagosome, and autolysosome 31. It often occurs during tumorigenesis and chemotherapy. Constructive autophagy can protect cancer cells from damage caused by chemotherapy to a certain extent, leading to drug resistance and refractory cancer 32. It has been reported that ATF4 is a critical signal for ER stress-induced autophagy. In this study, we observed that LC3-II accumulated in a dose-dependent manner after treatment with UA232 using western blotting and immunofluorescence. LC3 is widely used as an autophagy marker protein, and its level of LC3-II can characterize the number of autophagosomes 33. Additionally, we found that inhibition of ER stress by 4-PBA could restore the accumulation of LC3-II. This demonstrated that the activation of autophagy was triggered by ER stress in HeLa cells. To further study the function of autophagy in cell apoptosis, we used 3-MA to inhibit the formation of autophagosomes, and the results proved that autophagy played a protective role in HeLa cells.

However, it is worth noting that a remarkable increase of p62 was observed at the same time. p62 is a substrate of autophagy that can represent the smoothness of autophagic flux 34. The expression levels of LC3- II and p62 both increased, indicating that autophagy was increased but autophagic flux was blocked. Therefore, our study showed that UA232 is a potent autophagic flux inhibitor that increases cell sensitivity to chemotherapy. To investigate whether autophagic flux inhibition occurred because UA232 stopped the autophagosomes to fuse with lysosomes, we performed an immunofluorescence assay to detect the colocalization of LAMP2 and LC3. The results revealed that UA232 did not block the fusion of lysosomes and autophagosomes. However, there was a translocation of lysosomes from the nucleus to the plasma membrane. We further detected the different effects of UA232 on lysosomal functions to gain a full understanding of the possible mechanism by which UA232 inhibits autophagic flux. As we speculated, UA232, as lysosomal alkalinize-induced lysosome biogenesis and LMP, even induces the leakage of macromolecules inside the lysosomes which then causes lysosome-dependent cell death (LDCD).

Since lysosomes are crucial in degradation and recycling at the last stage of autophagy, lysosome inhibitors are often used as autophagy inhibitors. Chloroquine (CQ), hydroxychloroquine (HCQ), and amodiaquine-amodiaquine (AQ) are widely used as autophagy inhibitors for tumor therapy, which destroys lysosomal function and blocks autophagosomes to fuse with lysosomes 35, 36. However, according to previous research, the anti-tumor activity of using CQ or HCQ alone to block autophagic flux is very limited, and high-dose HCQ can only produce a moderate degree of autophagic flux obstruction in the clinic 37. Only when HCQ is combined with autophagy stimulation, can it significantly enhance the anti-tumor effect and reduce the chemotherapy dose. Therefore, combining ER stressors and lysosomal inhibitors clinically or developing compounds that can simultaneously trigger ER stress and lysosomal dysfunction can be used as effective strategies to kill tumor cells.

**Figure 8:**
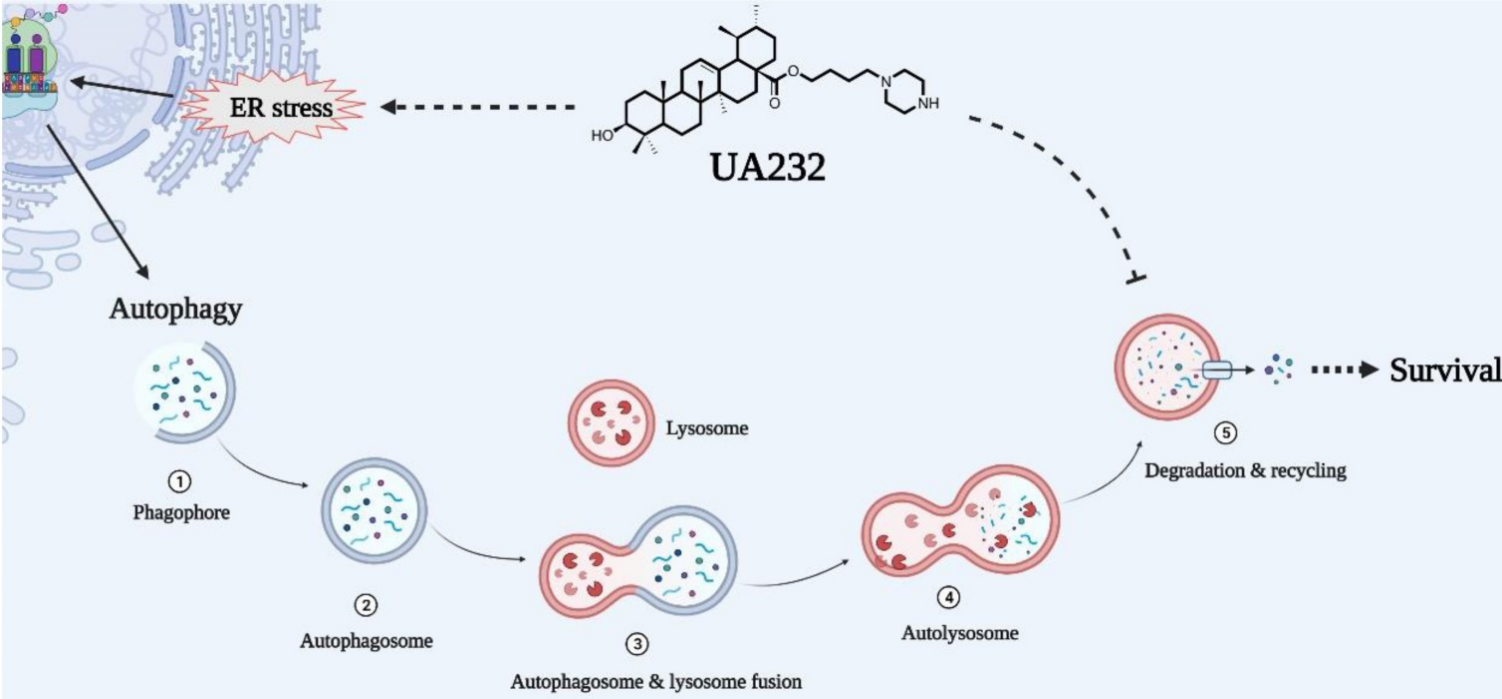
** Schematic representation of mechanisms of UA232 regulated apoptosis.** UA232 activates CHOP through the PERK-ATF4 signal pathway, which induces severe endoplasmic reticulum stress (ER stress) in tumor cells and promotes cancer cell apoptosis. In addition, UA232 damages the lysosome and triggers lysosomal membrane permeabilization (LMP), which leads to the leakage of macromolecular enzymes such as CTSB from the lysosome to the cytoplasm and induces lysosome-dependent cell death (LDCD) in the cell.

Overall, we report that UA232, as a weakly alkaline derivative of UA, has a strong anti-tumor activity *in vitro* and *in vivo*, which can be attributed to its influence on two main physiological processes, including its induction of severe UPR and lysosomal dysfunction. Moreover, autophagic flux, which serves as the homeostasis regulation of cells against insults, is impaired due to lysosomal alkalization and dysfunction. In other words, UA232 can not only induce cell apoptosis but can also make cells more sensitive to drugs by inhibiting autophagic flux (Fig. 8). Therefore, we believe that UA232 has the potential to be developed as an excellent anti-cancer agent for treating cervical cancer. This dual-pronged mechanism of drugs may be of great reference value for the treatment of refractory cancers and is worthy of further study.

## Supplementary Material

Supplementary figures.

## Abbreviations

UA: Ursolic acid
ER: Endoplasmic reticulum
UPR: Unfolded Protein Response
4-PBA: 4-Phenylbutyric acid
CTSB: Cathepsin B
CTSD: Cathepsin D
3-MA: 3-Methyladenine
TFEB: Transcription factor EB
LMP: Lysosomal membrane permeabilization
LDCD: lysosome-dependent cell death
