# Supplementary material for: Ursolic Acid Derivative UA232 Promotes Tumor Cell Apoptosis by Inducing Endoplasmic Reticulum Stress and Lysosomal Dysfunction
Source: Int J Biol Sci. 2022 Mar 21;18(6):2639–51. doi: 10.7150/ijbs.67166 (PMC8990475; doi:10.7150/ijbs.67166)
Supplement: Supplementary file 1 — Supplementary figures. [file ijbsv18p2639s1.pdf]

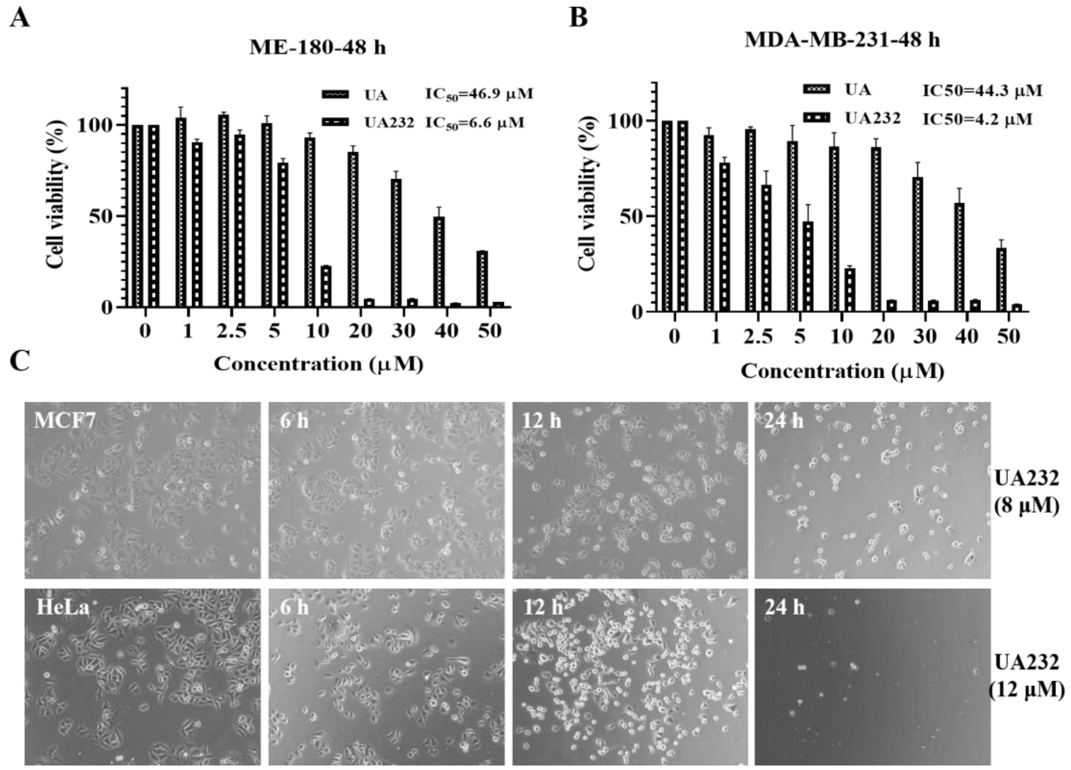

Figure S1. Effects of UA232 on ME-180, MDA-MB-231, HeLa, and MCF7 cell viability. The cell viability was determined by CCK8 assay in ME-180 **(A)** and MDA-MB-231 **(B)** cells treated with UA232 or UA (0-50 μM) for 48 h. **(C)** Cellular morphology of MCF7 or HeLa cells was detected after treatment with UA232 (8 or 12 μM) for 6,12, or 24 h.

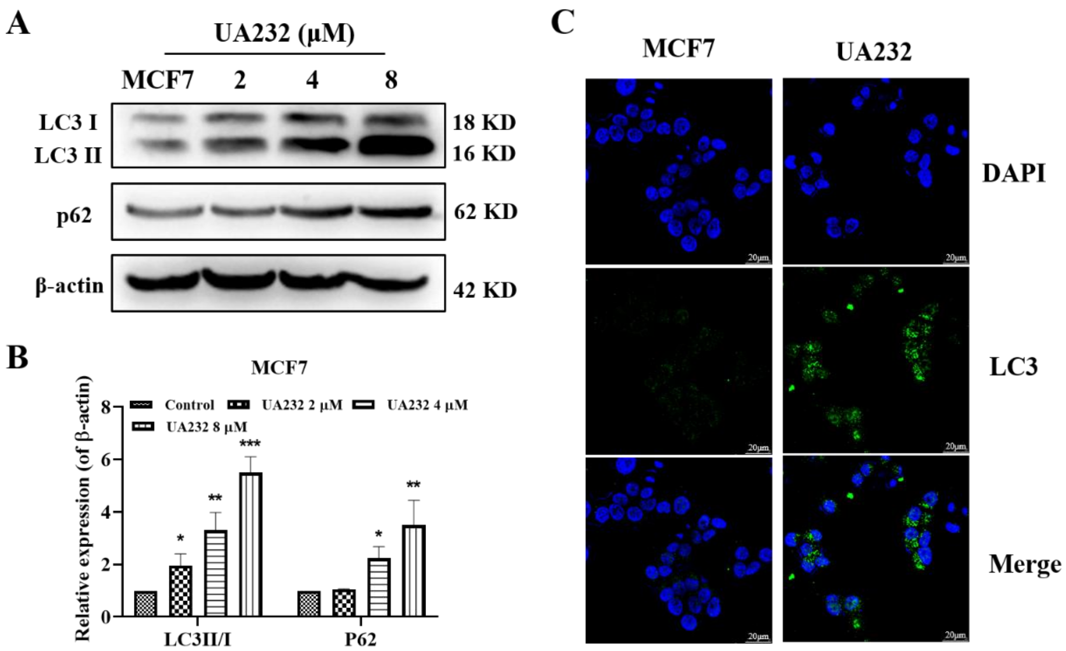

Figure S2. UA232 induces autophagy in MCF7 cells. (A) Changes in the expression of autophagy-related proteins in MCF7 cells treated with different concentrations of UA232 (2, 4, or 8 μM) for 12 h. **(B)** The quantitative data of Western blot analysis in panel A. **(C)** Immunofluorescence staining of LC3 in MCF7 cells treated with or without UA232 (4 μM), Nuclei are represented by blue (DAPI) staining, bar = 20 μm. $^{*}p < 0.05$, $^{**}p < 0.01$, $^{***}p < 0.001$ versus control.

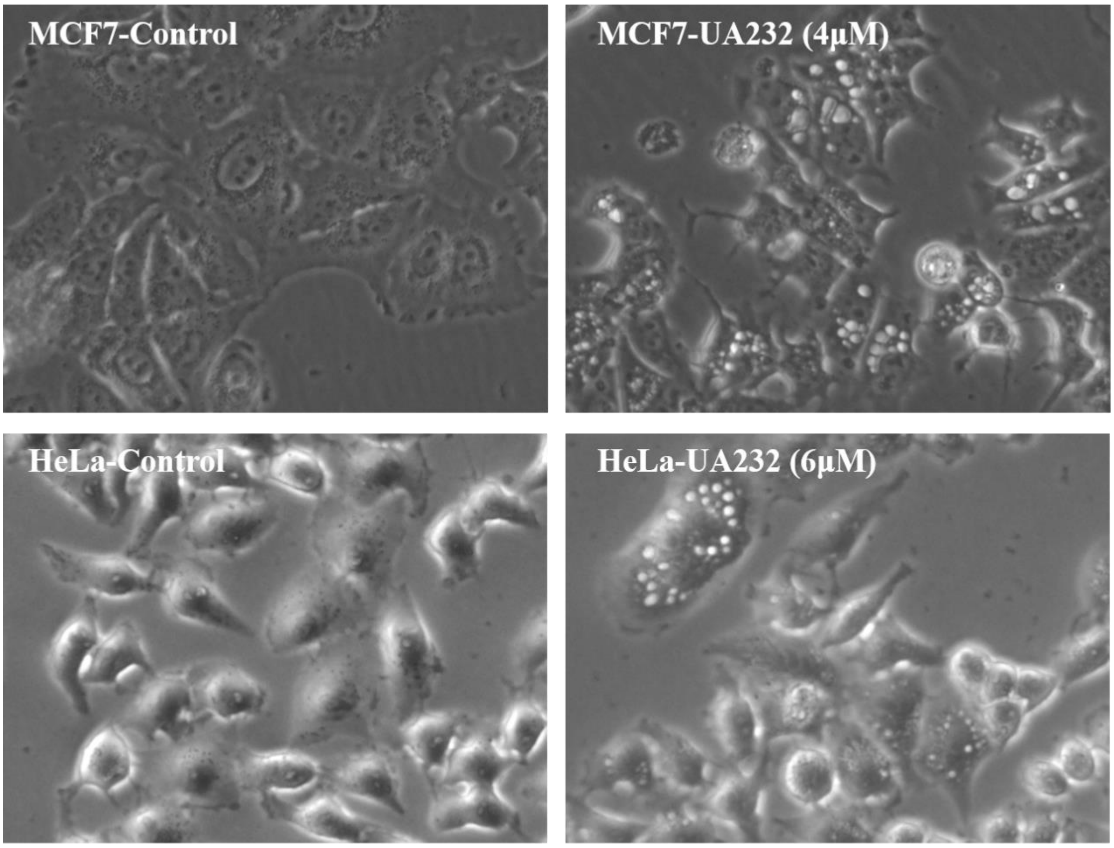

Figure S3. UA232 induces vacuolar changes in MCF7 and HeLa cells. Cellular morphology of MCF7 or HeLa cells was detected after treatment with UA232 (4 or 6 μM) for 12 h.

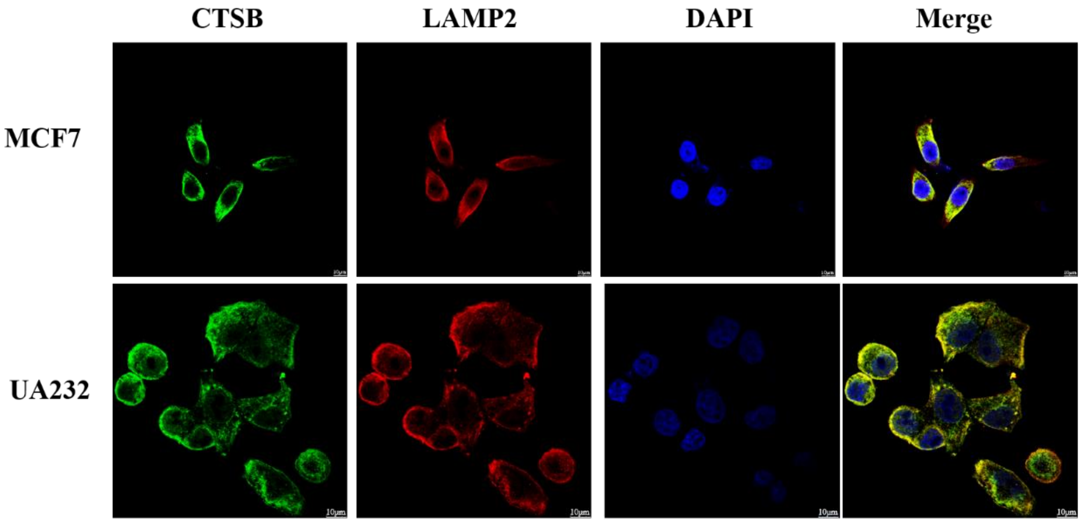

Figure S4. UA232 induces the release of CTSB from lysosomes in MCF7 cells. Immunofluorescence staining of CTSB (green) and LAMP2 (red) in MCF7 cells treated with or without UA232 (4 μM), bar = 5 μm.
